# Caliber of Rohon–Beard Touch-Sensory Axons Is Dynamic In Vivo

**DOI:** 10.1523/ENEURO.0043-25.2025

**Published:** 2025-05-21

**Authors:** Kaitlin Ching, Alvaro Sagasti

**Affiliations:** Department of Cell, Molecular, and Developmental Biology, University of California, Los Angeles, Los Angeles, California 90095

**Keywords:** axon, caliber, somatosensory, zebrafish

## Abstract

Cell shape is crucial to cell function, particularly in neurons. The cross-sectional diameter, also known as caliber, of axons and dendrites is an important parameter of neuron shape, best appreciated for its influence on the speed of action potential propagation. Many studies of axon caliber focus on cell-wide regulation and assume that caliber is static. Here, we have characterized local variation and dynamics of axon caliber in vivo using the peripheral axons of zebrafish touch-sensing neurons at embryonic stages, prior to sex determination. To obtain absolute measurements of caliber in vivo, we paired sparse membrane labeling with super-resolution microscopy of neurons in live fish. We observed that axon segments had varicose or “pearled” morphologies and thus vary in caliber along their length, consistent with reports from mammalian systems. Sister axon segments originating from the most proximal branch point in the axon arbor had average calibers that were uncorrelated with each other. Axon caliber also tapered across the branch point. Varicosities and caliber, overall, were dynamic on the timescale of minutes, and dynamicity changed over the course of development. By measuring the caliber of axons adjacent to dividing epithelial cells, we found that skin cell division is one aspect of the cellular microenvironment that may drive local differences and dynamics in axon caliber. Our findings support the possibility that spatial and temporal variation in axon caliber could significantly influence neuronal physiology.

## Significance Statement

Axon caliber directly influences how quickly neurons send messages to other cells and likely plays a role in neurons’ overall health. In the peripheral nervous system, where neurons cover particularly long distances, cell shape can determine whether an animal successfully executes behaviors such as escape responses. We found that axon caliber can vary between locations within the same cell and is highly dynamic. Taking these variations into account may allow neuroscientists to better estimate transmission speeds for cells in neural circuits. We observed that axon caliber is distorted when nearby skin cells change shape. Thus, cells not classically considered part of the nervous system can also contribute to caliber dynamics, broadening our view of axon caliber determinants.

## Introduction

The shape of a neuron's axons or dendrites is central to its function. A key characteristic of axon and dendrite shape is their cross-sectional diameter, also known as caliber. Axon caliber intrinsically influences the speed of action potential propagation (Sakaguchi et al., 1993; Kriz et al., 2000) and may also play a role in cell health and structural stability (Perge et al., 2012; Dollé et al., 2018).

**Figure 1.**
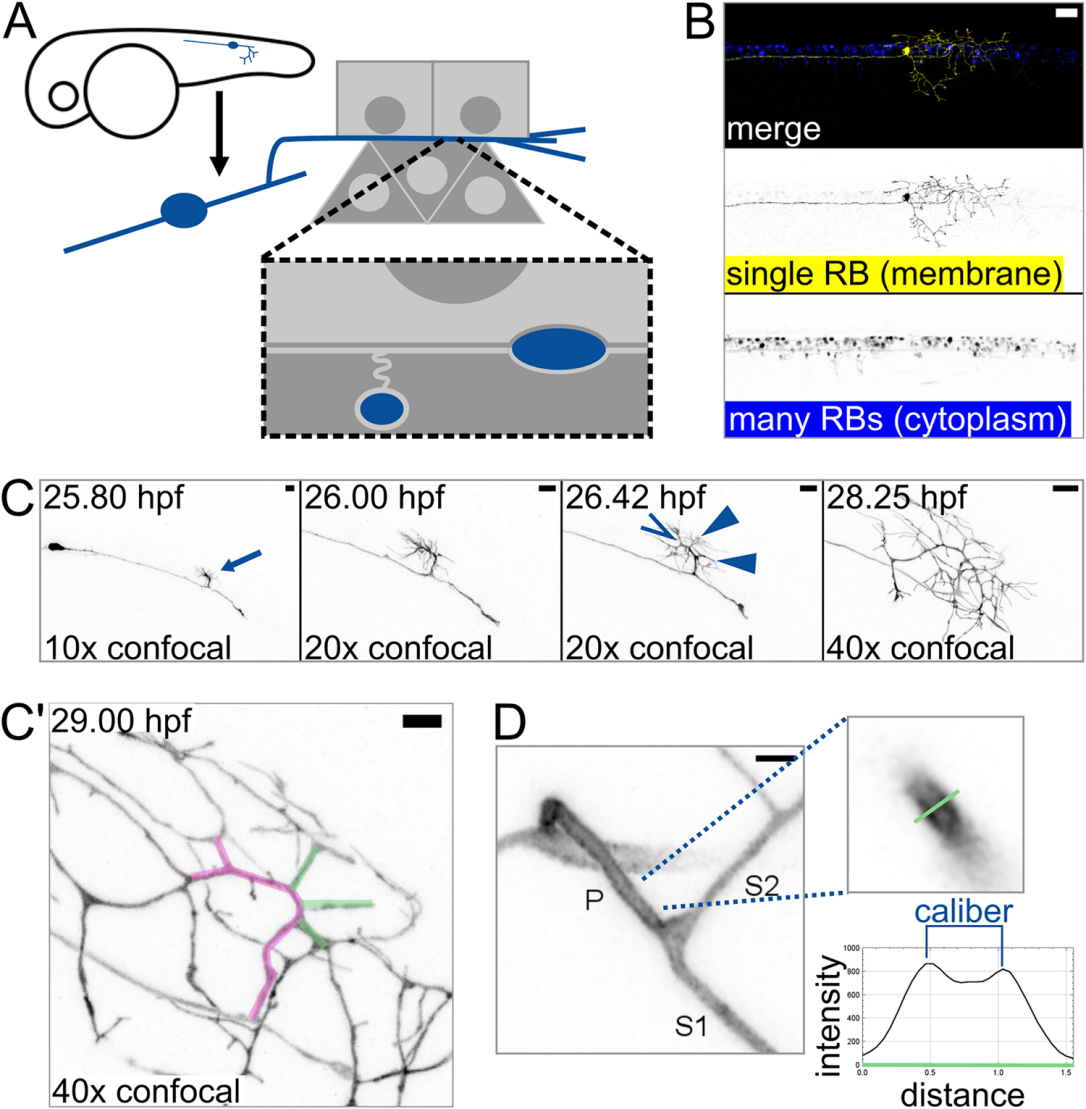
Axon branch development and caliber in sparsely labeled Rohon–Beard (RB) neurons. ***A***, Diagram depicting an RB neuron in an embryonic zebrafish. Light gray squares represent periderm (outermost) epithelial cells. Dark gray triangles represent basal epithelial cells, which ensheath some RB axons ∼2–3 dpf. ***B***, Example of embryonic zebrafish tail with sparse labeling showing one RB neuron expressing EGFP-CAAX among many RB neurons expressing cytoplasmic DsRed. Scale bar, 50 µm. ***C***, Illustrative images taken from time lapse of RB neuron development. Proximal branch point formed by collateral sprouting in 10 out of 10 time lapses. Arrow: formation of the main branch in the peripheral axon arbor. The 26.42 hpf panel shows multiple new branches that formed within minutes of each other. Open arrowhead: growth cone bifurcation event. Solid arrowheads: collateral sprouting events. Scale bar, 10 µm. ***C*’**, Proximal region of the axon arbor shown in ***B***. Magenta pseudocolored axon shows the main branch, which branched by growth cone bifurcation. Green pseudocolored axons show branches that were formed by subsequent collateral sprouting. Scale bar, 5 µm. ***D***, Method used for measuring caliber. RB neurons expressing EGFP-CAAX were measured by line scan (green line). Caliber was scored as the distance between intensity peaks, as illustrated in the example plot. P, primary branch, which extends from the spinal cord to the skin; S1, thicker secondary branch; S2, thinner secondary branch. Scale bar, 2 µm.

Rohon–Beard (RB) neurons are a type of touch-sensing neuron that develops in early embryonic zebrafish and project skin-innervating peripheral arbors with branched architecture (Fig. 1*A*; Wang et al., 2013; Katz et al., 2021; Shorey et al., 2021). RB peripheral arbors are afferent axons that detect somatosensory stimuli in the skin and relay messages to interneurons via central axons in the spinal cord, thus promoting behavioral responses.

The significance of axon caliber in cell function has been described for about a century (Hursh, 1939), and it is widely regarded as a cell-wide, static trait despite reports of variations in axon or dendrite caliber over space and time. Axon caliber can vary along the length of axons due to inherent biophysical properties (Griswold et al., 2024), myelination (Seidl, 2014), and mechanical insult (Pan et al., 2024). Dendrite caliber is also known to taper across branch points (Craig and Banker, 1994). Axon caliber has been observed to change over development (Seidl and Rubel, 2016; Bomont, 2021), aging (Metzner et al., 2022), and in response to target size (Voyvodic, 1989a,b) or activity (Sinclair et al., 2017; Nabel et al., 2024). These studies of caliber changes have primarily focused on changes to axon caliber over many days and assume that caliber undergoes little to no change on the timescale of hours. However, the cytoskeletal structures (Taylor et al., 2012; Costa et al., 2020; Wang et al., 2020) and cargoes (Greenberg et al., 1990; Costa et al., 2020; Wang et al., 2020) that can impact axon caliber are dynamic on the timescale of seconds to minutes, prompting us to ask how axon caliber and morphology change on shorter timescales.

Here, we characterized axon caliber in zebrafish RB neurons. We found that each single arbor, in vivo, can have a variety of axon calibers, varying within a segment and between sister segments. Axon calibers were dynamic on the timescale of minutes, and these dynamics changed over the developmental timescale of days. Finally, we found that the behavior of epithelial cells in the skin impacts sensory axon caliber, highlighting the complexities of the in vivo axon environment.

## Materials and Methods

### Fish care

Zebrafish (*Danio rerio*) adults were housed at 28.5°C with 13.5/10.5 h light/dark cycles. Embryos were raised at 28.5°C in water containing 0.06 g/L Instant Ocean salt mix (Spectrum Brands, AA1-160P) and 0.05% methylene blue (Thermo Fisher Scientific, 042771.AP).

### Injection for sparse labeling

To sparsely label RB neurons for observation of development and axon caliber, adult fish expressing Isl1[ss]:Gal4, UAS:dsRed (Sagasti et al., 2005) were crossed, and resulting embryos were allowed to develop for approximately 1 h after fertilization. When embryos reached the four-cell or eight-cell stage, a single cell in each embryo was injected with approximately 1–3 nl of plasmid containing the Gal4 effector, UAS:egfp-caax, diluted to 10–15 ng/µl in water, with or without phenol red (Sigma-Aldrich, P4758). After injection, embryos were raised as described above.

### Mounting embryos for microscopy

Unless otherwise indicated, embryos were immobilized on coverslips in 1.2% low-melt agarose dissolved in water (Thermo Fisher Scientific, BP165-25) and mounted with an equal volume of water containing 0.12 g/L Instant Ocean (Spectrum Brands, AA1-160P) and 0.4 mg/ml tricaine (MS-222, Western Chemical).

### Developmental time-lapse imaging

Embryos injected for sparse labeling were sorted at approximately 20 h postfertilization (hpf) to find animals with a single cell expressing EGFP-CAAX in the tail. Selected embryos were immediately dechorionated using forceps and anesthetized in approximately 0.2 mg/ml tricaine (MS-222, Western Chemical). Embryos were mounted as described above. A cut was made in the agarose posterior to the tail using a fly pin probe to allow room for growth. Embryos were imaged at 5 min intervals on a ZEISS laser scanning microscope (LSM) 800 with a stage heater at 28°C. Magnification was increased as needed by changing objectives to see branching. The most proximal branch point at approximately 28 hpf was identified and visually tracked back to the point of formation within the time lapse for scoring.

### Line scan and symmetry analysis

Embryos were injected for sparse labeling, as described above. Embryos with a distinct EGFP-CAAX–positive neuron in the tail were dechorionated using forceps and anesthetized in approximately 0.2 mg/ml tricaine (MS-222, Western Chemical). For this experiment, embryos were immobilized in 1.1–1.2% low-melt agarose on No.1 coverslips (Epredia, 12460S) to improve available working distance, and water used in initial anesthetization was added before sealing with a glass slide. The primary axon segment was identified as the one that exited the spinal cord and entered the skin. The most proximal branch point was imaged on a ZEISS LSM 880 with Airyscan deconvolution at 27–31 hpf. If two neurons or two arbors were present in the same animal or cell, the more posterior one was scored. Line scan analysis was performed using the Line and Plot Profile tools in ImageJ (Schindelin et al., 2012) to find intensity values along a line perpendicular to the center axis of the axon. Axon caliber was measured as the distance between the two peaks on the plot that corresponded to the plasma membrane. For symmetry analysis, axons were measured 3, 4, and 5 µm away from the branch point and averaged to find the caliber of the given segment. The caliber of secondary branches was normalized by dividing by the caliber of the corresponding primary branch. We defined symmetry as S2/S1, which is equivalent to the normalized S2 caliber divided by the normalized S1 caliber.

### Caliber dynamic time-lapse imaging

Embryos were injected for sparse labeling, as described above. Approximately 24 hpf, embryos were mounted as described above. The proximal region of the axon arbor was imaged on a ZEISS LSM 980, heated to approximately 28°C, and processed with Airyscan Joint Deconvolution at a maximum of five iterations. Time lapses with 5 min intervals were started at 28–31 hpf and acquired until the axon drifted out of the field of view, typically 1–2 h. After imaging, embryos were recovered from the mounting agarose using forceps and a probe and grown an additional 24 h under normal conditions (described above), with the addition of 0.2 mM 1-phenyl 2-thiourea (PTU, Sigma-Aldrich, P7629) in the water. At 52–55.5 hpf, embryos were mounted and imaged in the same manner as on the first day. For scoring, axon segment calibers were measured 3 µm from a branch point at every frame of the time lapse, and segments used were each separated by at least one branch point. If the same segments could be identified on the second day, caliber was scored at the same locations for every frame of the time lapse.

### Dual color time-lapse imaging of cell division

To observe axons near dividing cells, transgenic embryos expressing Isl1[ss]:lexA, lexAop:mRuby-caax and tp63:Gal4VP16; UAS:egfp-plcδ-ph (Rasmussen et al., 2015) were mounted as described above and imaged on a ZEISS LSM 980, heated to approximately 28°C, and processed with Airyscan Joint Deconvolution at a maximum of five iterations. Basal skin cells in a rounded state were identified by eye when embryos were 28–31 hpf and immediately imaged by time lapse with 5 min intervals, until daughter basal cells appeared flattened for at least two time points. Axon caliber was measured by line scan analysis, as described above, at 1 µm intervals, excluding regions <3 µm from a branch point. The border-to-border length of axons was measured using the Line tool in ImageJ (Schindelin et al., 2012). Orthogonal projections were generated in ZEN Blue (ZEISS).

### Plotting

All dot plots were created in R. Code can be found at github.com/katieching/CaliberAnalysis. Histograms, dynamics, and pearling plots were created using Microsoft Excel.

### Experimental design and statistical analysis

For descriptive analyses, an appropriate size for the data set was chosen in advance, and images were collected in experimental batches until the predetermined size was exceeded. For statistical comparisons, a small data set was initially collected, and a power analysis was performed to determine the data set size needed to reach a *p*-value of <0.05. The data set was collected and analyzed before performing final statistical analyses. Data were excluded as necessary for technical reasons, namely, image quality that was too poor for line scan analysis, segments where branch points were closer together than the distance indicated for the experiment (e.g., <5 µm for symmetry analysis), images in which the primary branch caliber was below the limit of resolution for the system (Figs. 3, 4), and axons in which >20% of measurements at the 1 d postfertilization (dpf) time point were below the limit of resolution for the system (Figs. 5, 6). Significance of differences in paired data was evaluated by paired permutation test in R. Differences between measurements treated as populations were evaluated by Mann–Whitney *U* test. Analyses performed in R can be found at github.com/katieching/CaliberAnalysis. Where appropriate, estimation statistics were calculated using www.estimationstats.com (Table 1). The sex of animals included in the study was not considered because experiments occurred prior to sex determination.

**Table 1.** Statistical table

	Corresponding figure	Data structure	Type of statistical test	Effect size and confidence interval (CI) or cutoff value
a	Figure 3*B* (comparing S1 with P)	Unknown	Paired mean difference	−0.167 µm with 95% CI (−0.207, −0.126)
b	Figure 3*B* (comparing S2 with P)	Unknown	Paired mean difference	−0.173 µm with 95% CI (−0.217, −0.134)
c	Figure 3*B* (comparing S2 with S1)	Unknown	Paired mean difference	−0.00617 µm with 95% CI (−0.0532, 0.0451)
d	Figure 3*C*	S1/S2 has a uniform distribution	Linear regression	*R*^2^ > 0.5 cutoff
e	Figure 5, *G* and *H*	Unknown	Linear regression	*R*^2^ > 0.5 cutoff
f	Figure 6*C* (comparing 2 with 1 dpf)	Unknown	Paired permutation test (in text) and paired mean difference (right)	−8.09% with 95% CI (−13.1, 0.223)
g	Figure 7*C* (on dividing cell vs neighbor1)	Not normally distributed	Mann–Whitney *U* test (in text) and paired mean differences (right)	0.0484 µm with 95% CI (0.0123, 0.103)
h	Figure 7*C* (on dividing cell vs neighbor2)	Not normally distributed	Mann–Whitney *U* test (in text) and paired mean differences (right)	Effect size of 0.0647 µm with 95% CI (0.0253, 0.124)
i	Figure 7*C* (on neighbor1 vs neighbor2, control)	Not normally distributed	Mann–Whitney *U* test (in text) and paired mean differences (right)	Effect size of 0.014 µm with 95% CI (0.0355, −0.0168)
j	Figure 8*D* (on dividing cell, round vs flat)	Unknown	Paired permutation test (in text) and paired mean difference (right)	Effect size of 0.0256 µm with 95% CI (0.0578, −0.0116)
k	Figure 8 (on nondividing neighbor, control)	Unknown	Paired permutation test (in text) and paired mean difference (right)	Effect size of 0.0013 µm with 95% CI (0.034, −0.027)

Additional statistics for quantitative data and corresponding figure panels.

## Results

### Rohon–Beard (RB) neurons display a variety of calibers

RB neurons are touch-sensing neurons that differentiate and become functional within 2 d of development in embryonic zebrafish and other anamniotes (Katz et al., 2021). The peripheral axons of RB neurons can be identified by transmission electron microscopy (TEM) as membrane-bound ovals between the outermost (periderm) skin cells and lower (basal) skin cells in cross section (O’Brien et al., 2012). Using existing TEM data sets (Faas et al., 2012; O’Brien et al., 2012), we identified peripheral axons of RB neurons and trigeminal neurons, closely related sensory neurons that perform the same touch-sensing function in the head. By measuring the short axis of these axon cross sections, we found that calibers ranged from approximately 0.084–1.459 µm. This wide range prompted us to ask if axon caliber varies within each cell or only between cells, perhaps by sensory subtype (Gau et al., 2013; Palanca et al., 2013; Tuttle et al., 2024).

To determine if axon caliber can vary within an axon arbor, we sought to measure caliber at consistent locations across many intact cells. To enable absolute measurements of axon caliber, we developed an imaging protocol that paired sparse labeling with super-resolution microscopy. Using transient transgenesis, we generated embryos that expressed a membrane-localized fluorescent protein (EGFP-CAAX) in a single RB neuron in the tail (Fig. 1*B*). We performed time-lapse confocal microscopy starting shortly after RB neuron differentiation (20 hpf) and confirmed that branches of the peripheral axon arbor form by growth cone bifurcation as well as collateral sprouting (Sagasti et al., 2005; Haynes et al., 2022), resulting in a highly branched axon arbor within hours of skin entry. To identify a consistent area of measurement, we observed the most proximal branch point in the peripheral arbor at 27–30 hpf and found that it typically forms by collateral sprouting (10 out of 10 cells; Fig. 1*C*,*C*’; Movie 1). In these images, some branches of each arbor appeared to be thick while others appeared to be thin (Fig. 1*D*). This observation suggested that axon caliber can vary within each cell and supports the use of this location and technique for studying axon caliber variation.

**Movie 1.** Time-lapse microscopy of axon branch formation in RB neurons. Confocal time-lapse microscopy of RB neuron development in the embryonic zebrafish tail. The main branch of the peripheral axon arbor grows out of the developing spinal cord and into the skin. Shortly thereafter, many branches form by growth cone bifurcation and collateral sprouting. Pseudocoloring in the final frames highlights proximal arbor, and arrows indicate branch points that were formed by collateral sprouting events at the times indicated. Scale bar, 5 µm. [View online]

### Caliber varies along the length of an axon segment

**Figure 2.**
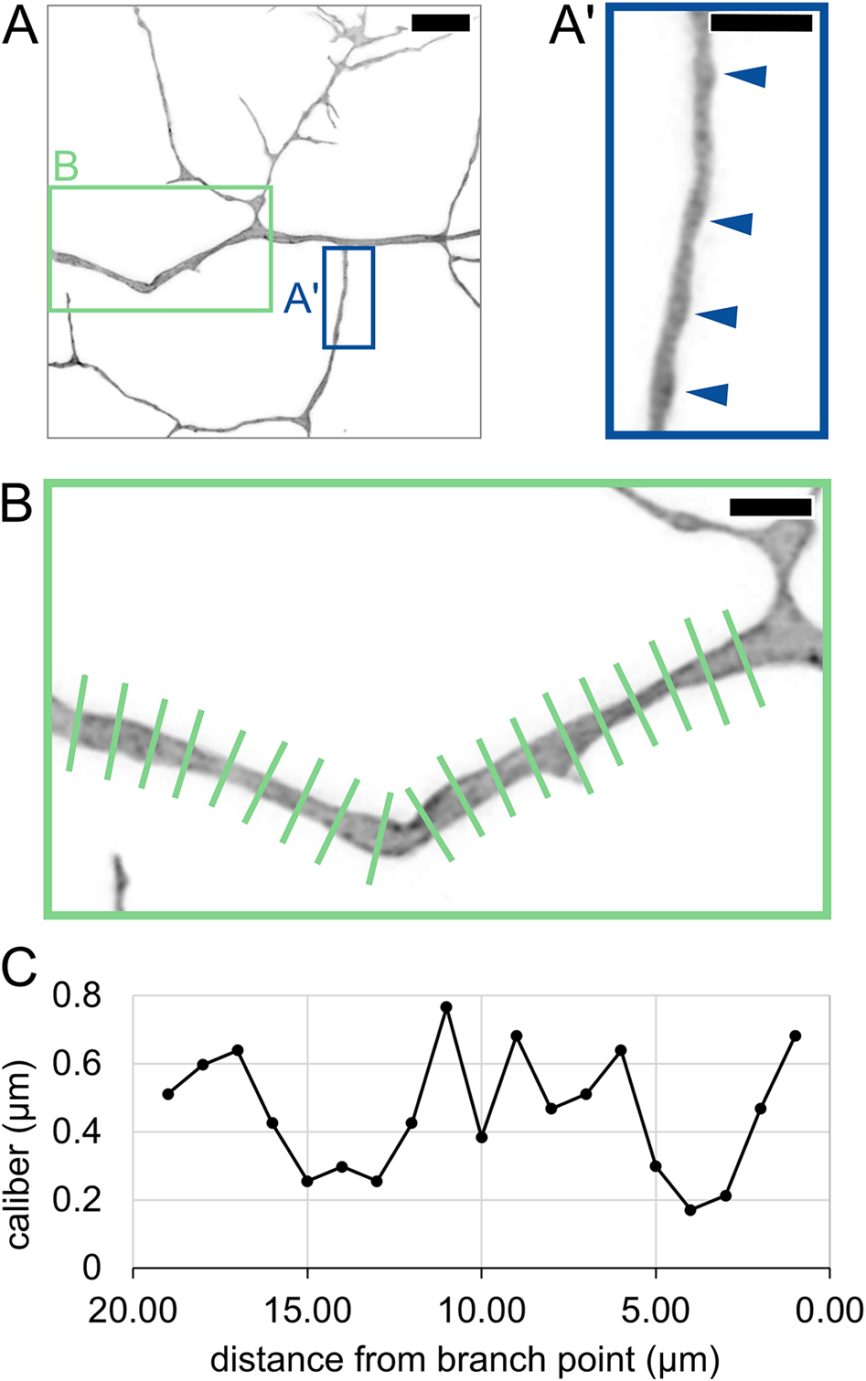
Lengthwise variation in axon caliber. ***A***, Image of an RB axon arbor at 29.00 hpf. Blue box shows location of inset in ***A*’**. Green box shows location of inset in ***B***. Scale bar, 5 µm. ***A*’**, Inset showing axon branch with pearled morphology. Arrowheads: thicker regions of axon, resembling “pearls” previously described in mammalian neurons. Scale bar, 2 µm. ***B***, Long axon segment measured by line scan at 1 µm increments. Scale bar, 2 µm. ***C***, Plot of caliber showing variation along the length of axon segment in panel ***B***.

Previous studies have shown that axons are not cylindrical but vary in caliber along their lengths (Greenberg et al., 1990; Bar-Ziv et al., 1999; Pullarkat et al., 2006; Griswold et al., 2024). This phenomenon is sometimes referred to as pearling or varicose morphology. To determine if pearling is observed in live animals, we labeled individual RB neurons and measured axon caliber at 1 µm increments along the length of a segment, shortly after arbor formation began (29 hpf; Fig. 2*A*,*B*). Variations in axon caliber were visible along the length of all resolvable axon segments, as has been described in mammalian neurons (Greenberg et al., 1990; Griswold et al., 2024). In some instances, axons had distinct pearls (Fig. 2*A*’) and, in others, more subtle thick and thin stretches without distinct inflection points (Fig. 2*C*). These observations of lengthwise caliber variation confirm that fish and mammalian neurons share similarities in local axon morphology in vivo.

### Sister axon branch calibers are largely uncorrelated with each other

**Figure 3.**
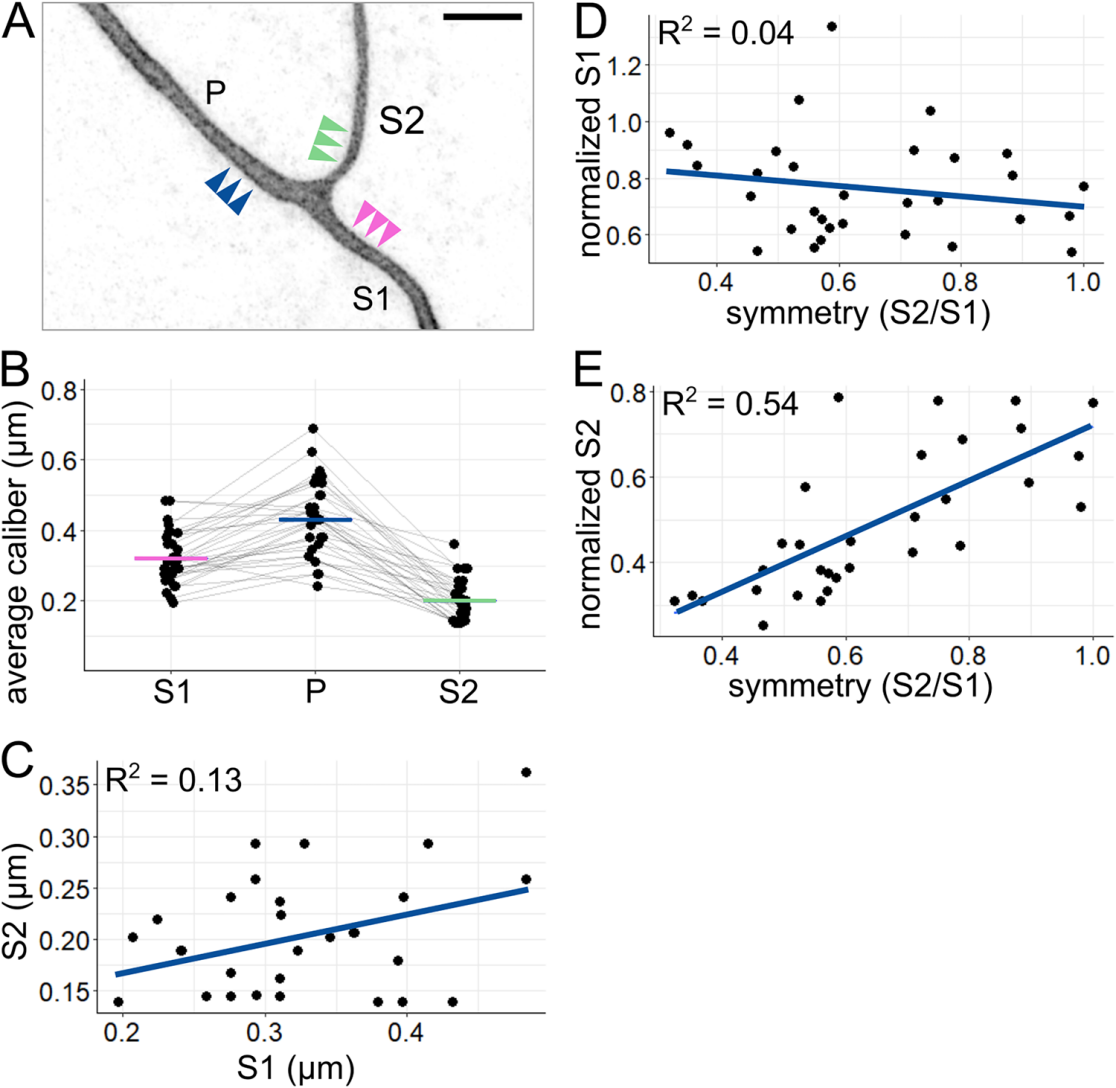
Comparison of axon segments within each arbor. ***A***, Example image of proximal RB axon arbor. Arrowheads indicate approximate location of line scans for average caliber measurement. P, primary branch; S1, thicker secondary branch; S2, thinner secondary branch. Scale bar, 5 µm. ***B***, Plot of average axon calibers at the proximal branch point. Gray lines connect data points from the same neuron. Horizontal lines: mean values with colors corresponding to branch categories shown in panel ***A***. *n* = 31 neurons. ***C***, Plot comparing caliber of sister axon segments shown in panel ***A***. ***D***, Plot of branch symmetry versus normalized S1 caliber (S1/P). ***E***, Plot of branch symmetry versus normalized S2 caliber (S2/P). Blue lines in ***C***, ***D***, and ***E*** show linear regression.

Next, we asked how axon caliber varies between different axon segments within a single cell. We labeled individual RB neurons and measured axon caliber near the branch point most proximal to the cell body in 27–31 hpf embryos (Fig. 3*A*). The axon segment coming from the spinal cord was designated the primary axon (*P*), and the axon segments distal to the branch point were designated secondary axons (sister branches designated S1 and S2, for the thicker and thinner segments, respectively). Each segment was measured at three locations (3, 4, and 5 µm from the branch point), which were averaged to estimate the caliber of that axon segment. By measuring these locations, we observed that axon segments of different calibers exist within the same RB neuron and that secondary branches were typically thinner than the primary branch from which they arose (Fig. 3*B*, *p* = 0.43 µm, S1 = 0.32 µm, S2 = 0.20 µm; Table 1, a–c). These data confirmed that the average caliber of axons is not uniform across the cell.

Because sister segments, S1 and S2, often have different calibers, we sought to assess the relationship between them. We considered two hypotheses: (1) despite variations, each neuron has characteristically thick or thin calibers, and thus S1 and S2 have positively correlated calibers, or (2) S1 and S2 compete for cellular material, and thus their calibers are negatively correlated. To distinguish between these two hypotheses, we compared the S1 and S2 caliber for each neuron. Direct comparison showed a weak relationship between S1 and S2 caliber (Fig. 3*C*, *R*^2^ = 0.13). This weak correlation suggests that neither hypothesis is strongly supported.

In keeping with this interpretation, symmetry across the branch point was weakly correlated with normalized S1 caliber (Fig. 3*D*, *R*^2^ = 0.04), which we would expect to be strongly correlated if branches compete for cellular material to increase caliber. On the other hand, symmetry is more strongly correlated with normalized S2 caliber (Fig. 3*E*, *R*^2^ = 0.54), suggesting that symmetry is achieved by the thinner branch being thicker, unrelated to the caliber of the S1 branch.

### RB axons taper

For an RB neuron to function properly, the cell needs electrical signals to travel efficiently. At the time points measured in Figure 3, the arbor was just a few hours old, functional, and continuing to expand. Already, optimal RB neuron function of transmitting sensory signals from the periphery requires efficient propagation of electrical signals from distal to proximal axon segments (toward the soma). Although the peripheral arbor of RB neurons transmits these afferent signals, as dendrites typically do, we refer to the peripheral arbor as an axon due to its uniformly plus-end-out microtubule organization (Shorey et al., 2021). Tapering is often considered a characteristic unique to dendrites (Craig and Banker, 1994; Jan and Jan, 2010), though more recent analyses suggest that branched axons may taper as well (Desai-Chowdhry et al., 2022).

**Figure 4.**
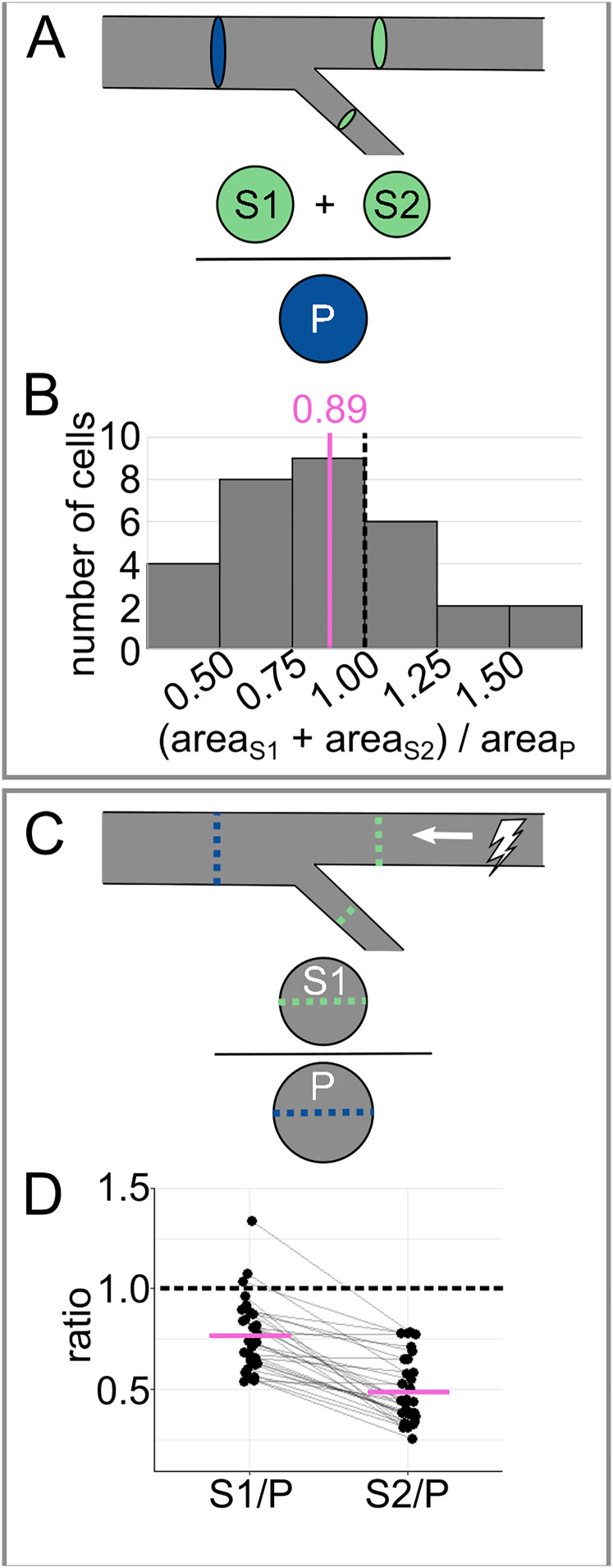
Analysis of tapering across the most proximal branch point. ***A***, Diagram of tapering analysis in panel ***B***. Combined cross-sectional area of secondaries (S1 and S2) is divided by the cross-sectional area of the primary (P) to evaluate tapering. ***B***, Histogram of cross-sectional area ratios for data shown in Figure 1. ***C***, Diagram of tapering analysis in panel ***D***. Caliber of each secondary (S1 or S2) is divided by the caliber of the primary (P) to estimate the impact of tapering on action potential conduction velocity. ***D***, Plot of caliber ratios for data shown in Figure 1. Gray lines connect data points from the same neuron. In panels ***B*** and ***D***, dotted black lines mark reference value 1.0 (no tapering), and magenta lines mark mean values.

To facilitate comparison of RB axon branching to other systems, we assessed tapering. First, we calculated the cross-sectional area of each primary and secondary branch $\left(\mathrm{area}=\pi \;*\;{\left(\frac{1}{2}\mathrm{caliber}\right)}^{2}\right)$. We compared the primary cross-sectional area with the sum of the secondary cross-sectional areas (Fig. 4*A*). On average, the ratio of the combined secondary cross-sectional areas to the primary cross-sectional area was <1 (mean of $\frac{\mathrm{are}a_{S1}+\mathrm{are}a_{S2}}{\mathrm{are}a_{P}}=0.888$, Fig. 4*B*), meaning that the RB peripheral arbor tapers in area.

Tapering can influence the health and function of a neuron in many ways, some of which scale with area and some of which scale with radius (Craig and Banker, 1994; Desai-Chowdhry et al., 2022). Hence, we also compared the radius scaling ratio between secondary branches and their corresponding primary branches ( $\frac{r_{S}}{r_{P}}$, Fig. 4*C*). The average ratio for the thicker branch was much larger than for the thinner sister branch (mean $\frac{S1}{P}\;=\;0.77\;\pm 0.033\;\mathrm{SEM}\;$and mean $\frac{S2}{P}=0.49\;\pm 0.030\;\mathrm{SEM}$, Fig. 4*D*). In evaluating a branched system, some scaling rules hold for the population average, even when branches are asymmetric (Brummer et al., 2017). Hence, to evaluate the function of the arbor more generally, we combined the measurements for the two branches and found that the average ratio (mean $\frac{S}{P}\;=\;0.629\;\pm \;0.0282\;\;\mathrm{SEM}$) was similar to what is predicted for a system that optimizes for power dissipation rather than time delay (Desai-Chowdhry et al., 2022).

Here, we refer to the RB peripheral arbor as an axon due to its plus-end-out microtubule organization (Shorey et al., 2021). Therefore, our observations demonstrate that it is possible for neuronal processes with an axon-like cytoskeletal organization to taper.

### Axon caliber is highly dynamic

**Figure 5.**
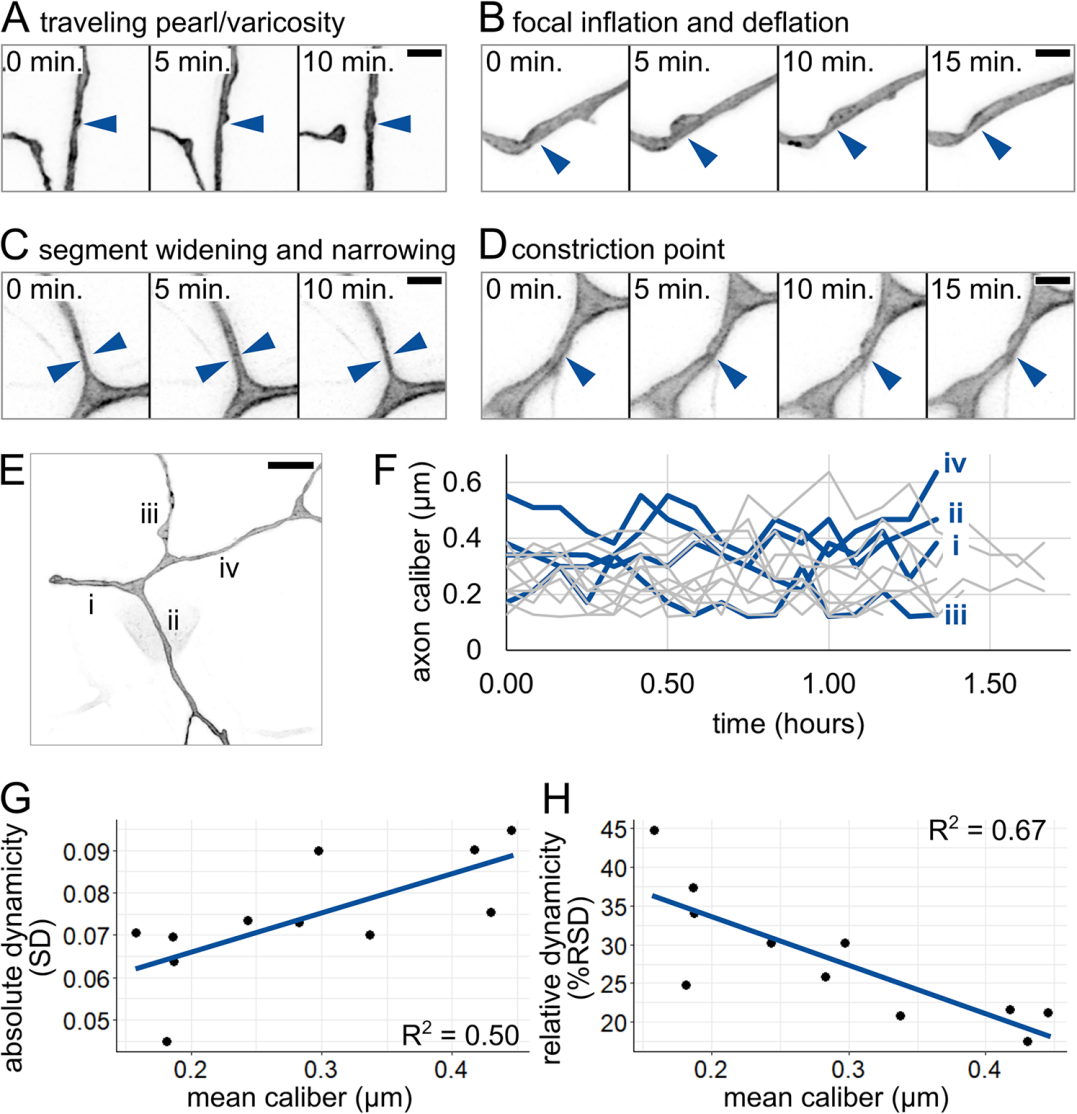
Qualitative and quantitative analysis of caliber dynamics. ***A***, Example of a possible traveling pearl. Short distance traveled allowed for acquisition despite 5 min intervals. Arrowhead: initial location of pearl. ***B***, Example of focal inflation and deflation event. Puncta containing EGFP-CAAX accumulate during inflation and are seen exiting during deflation. Arrowhead: location of event. ***C***, Example of segment widening and narrowing. Arrowhead pairs denote width at *t* = 5 min. ***D***, Example of constriction point. Arrowhead: location of event. Scale bars: ***A–D***, 2 µm. ***E***, Image of axon arbor in the proximal region. Different segments are labeled from proximal to distal: i–iv. Scale bar, 5 µm. ***F***, Plot of axon caliber dynamics, *n* = 11 axon segments, *N* = 5 fish. Blue lines: axon segments shown in panel ***E***. Gray lines: all other axon segments measured. ***G***, Plot of mean caliber versus absolute dynamicity (SD) for all axons shown in panel ***F***. Mean caliber is the average at the given location across all time points. ***H***, Plot of mean caliber versus relative dynamicity (%RSD = (SD/mean caliber) × 100) for all axons shown in panel ***F***. In panels ***G*** and ***H***, blue lines denote linear regression.

Having documented variability in axon caliber across locations within each RB neuron, we wondered how calibers change with time. To investigate caliber dynamics, we performed time-lapse imaging in the proximal region of the tail-innervating axon arbors at 28–31 hpf (Movie 2). Axon caliber changed in several qualitatively different ways over time. We categorized these dynamic behaviors into four categories (Movie 3): (1) a traveling pearl, instances of bubble-like regions moving along an axon (Fig. 5*A*); (2) focal inflation and deflation, in which a pearl appears and disappears over the course of multiple time points (Fig. 5*B*); (3) segment widening and narrowing, in which an entire segment thickens and thins between time points (Fig. 5*C*); and (4) a constriction point that appears and disappears, sometimes repeatedly (Fig. 5*D*). This variety of dynamic behaviors suggests that several distinct processes likely contribute to axon caliber dynamics.

**Movie 2.** Time-lapse microscopy of the peripheral RB axon. Super-resolution time-lapse microscopy of the peripheral axon arbor showing the most proximal branch points. Frequent and rapid changes in axon caliber occur within the approximately 1 h captured prior to drift. Time interval, 5 min. Scale bar, 5 µm. [View online]

**Movie 3.** Types of caliber fluctuations in RB axons. Super-resolution time-lapse microscopy of peripheral axon arbors showing examples of qualitatively different fluctuations in axon caliber. Time interval, 5 min. Scale bar, 2 µm. [View online]

Axon caliber is known to be altered by changes in target cells (Voyvodic, 1989a; Sinclair et al., 2017; Nabel et al., 2024) or myelination (Seidl, 2014; Walker et al., 2019; Ciocanel et al., 2020), but these developmental processes occur on different timescales from the dynamics we observed with intervals of 5 min (Fig. 5*A*–*D*). To assess caliber dynamics quantitatively, we selected several locations within the proximal region of each neuron, each 3 µm away from a branch point, and measured axon caliber at that location over the course of imaging (Fig. 5*E*). Axon caliber was highly dynamic (Fig. 5*F*), with standard deviations ranging from 0.045 to 0.095 µm and could vary with a range of up to fourfold at the same location in <2 h. Hence, axon caliber and local morphology may be more dynamic than has been widely appreciated.

Next, we asked if thin or thick axon segments are more dynamic. First, we assessed absolute dynamicity by comparing average caliber with the standard deviation (SD) of measurements observed during the time lapse (Fig. 5*G*). We found that SD and axon caliber correlated positively, suggesting that thicker axons experienced larger or more fluctuations in caliber. A possible caveat for our measurements is that the lower dynamicity could be due to thin axons spending more time below the limit of resolution. To determine if this was the case, we also assessed relative dynamicity. We calculated the relative standard deviation (RSD) for each location by dividing the standard deviation by the average caliber across the movie. Comparing dynamicity across segments revealed that caliber and RSD were negatively correlated, meaning that thick axons were less dynamic than thin axons (Fig. 5*H*). If differences in absolute dynamicity were due to resolution limitations, we would expect to see that the relative dynamicity of the thinnest axons is also low. Instead, thin axons had high relative dynamicity, indicating that, if anything, we may have underestimated the dynamicity of thin axons. Together, these results suggest that thick axons have more or larger absolute fluctuations in caliber, but the fluctuations experienced by thin axons are more dramatic when compared with their average caliber.

### Caliber dynamicity changes over development

**Figure 6.**
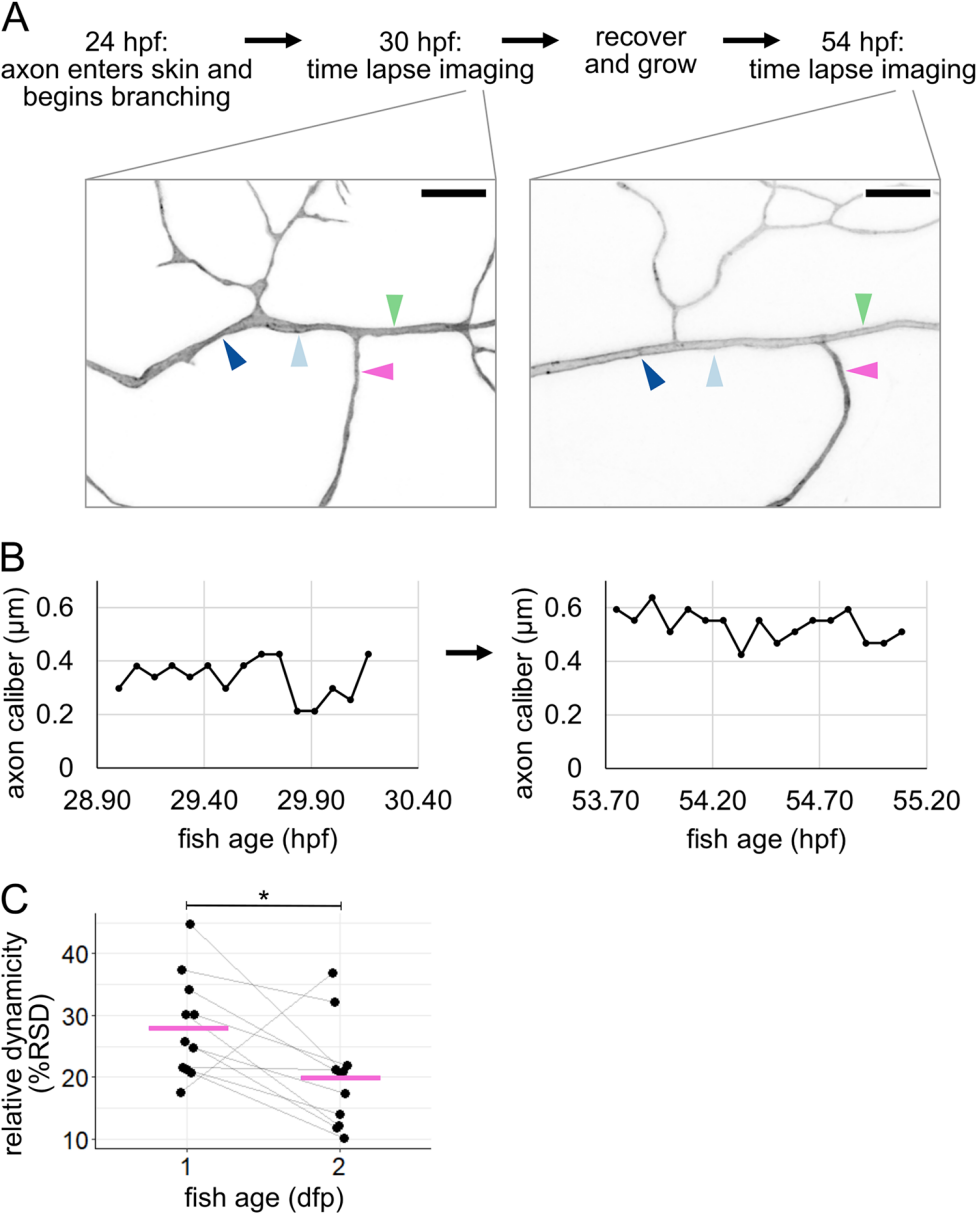
Tracking caliber dynamics over development. ***A***, Experimental workflow with example images (same neuron as Fig. 2). Arrowheads: locations for line scan measurements. Each location is 3 µm from a branch point and separated from other locations by at least one branch point. Scale bar, 5 µm. ***B***, Representative example plots of caliber dynamics for the same segment at 1 and 2 dpf. ***C***, Plot of relative dynamicity (%RSD) at 1 and 2 dpf (same neurons as Fig. 5), *n* = 11 neurons, *N* = 5 fish. Gray lines connect data points from the same neuron. Magenta lines: mean values. *p < 0.05.

Because we observed caliber to be highly dynamic early in the life and function of RB neurons, we sought to determine if caliber continues to be dynamic later in development. To do this, we recovered embryos after imaging at 28–31 hpf and allowed them to continue developing for an additional 24 h. These fish were remounted at 52–55.5 hpf and imaged by time-lapse microscopy again. Although all cells had changed shape and axon arbors had grown, often the locations imaged on the first day could be identified, imaged, and measured again on the second day (Fig. 6*A*,*B*). We found that all resolvable axons continued to be dynamic. However, dynamicity measured as %RSD decreased from the first to the second day in most axon segments (10 out of 11 axon segments decreased. Mean %RSD = 28% at 1 dpf and 20% at 2 dpf, *p* = 0.036, Fig. 6*C*; Table 1, f). Because some axon segments got thicker (*n* = 6 out of 11) and some got thinner (*n* = 5 out of 11), this decline in relative dynamicity is not simply a product of axons becoming universally thicker. Instead, this dynamicity likely reflects developmental changes that occur in the cell and tissue.

### Cellular microenvironment can impact axon caliber

Based on the qualitative variety of caliber dynamics we observed on the minutes timescale (Fig. 5*A*–*D*), we hypothesized that there are multiple contributors to caliber dynamics. Cell-intrinsic effectors that may cause axon caliber to be dynamic on short timescales, such as cargo transport and contraction of the membrane periodic skeleton (MPS), have been described by others (Costa et al., 2020; Wang et al., 2020). However, another contributor is the cellular microenvironment. Mechanical force at the soma has been shown to influence axon and dendrite morphology (Pan et al., 2024). We hypothesized that, in RB peripheral axons, caliber changes could be influenced locally, including by contact with surrounding epithelial cells. Because RB axons are embedded in actively developing skin, they are subject to local deformation caused by morphological changes in surrounding epithelial cells. Specifically, RB peripheral axons grow between two epithelial cell layers, the periderm and basal cells, each of which undergoes rapid expansion at these developmental stages (Fig. 1*A*; O’Brien et al., 2012).

**Figure 7.**
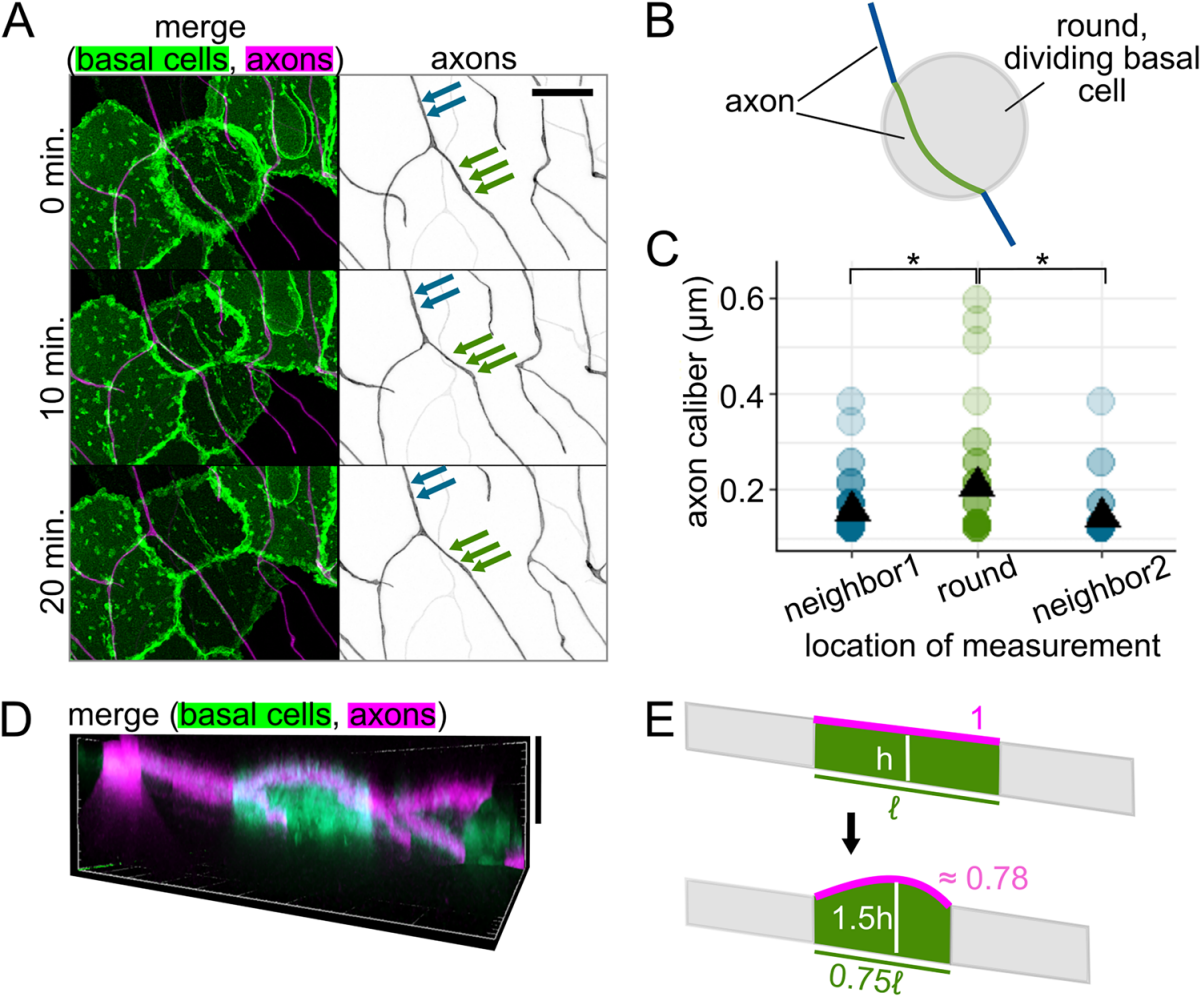
Axon caliber on dividing basal epithelial cells. ***A***, Representative images of time lapse showing an RB axon adjacent to a dividing basal cell. Basal cells express EGFP-PLCδ-PH. RB neurons express mRuby-CAAX. Green arrows: example measurement locations for an axon on a dividing basal cell. Blue arrows: example measurement locations for an axon on a nondividing neighbor cell. Scale bar, 10 µm. ***B***, Diagram of an axon on a dividing basal cell that is rounded while undergoing mitosis. Green line: axon segment in contact with dividing basal cell. Blue lines: axon segments on neighboring, nondividing basal cells. ***C***, Plot of axon caliber measurements grouped by location, *n* = 114 measurement locations, *N* = 4 axons/fish. Green dots: caliber of axon on a dividing basal cell. Blue dots: caliber of axon on neighboring cells to one side (neighbor1) or the other (neighbor2) of the dividing basal cell. Black triangles: mean value. *p < 0.05. ***D***, Orthogonal projection of a dividing basal cell, which expresses EGFP-PLCδ-PH and is highlighted against nondividing basal cells due to a variegated expression pattern. An RB axon is in contact with its apical surface, which is rounded upward. Vertical scale bar, 10 µm. ***E***, Diagram comparing axon path length on a rounded, dividing basal cell to its flattened state as an approximation of how basal cell shape changes may impact tension on the axon. Magenta: approximated, theoretical axon length, estimated as the longest distance across the apical surface in an orthogonal projection. Approximate arc length was calculated based on height and length estimates in orthogonal projections. ℓ, original length of basal cell; h, original height of basal cell.

To determine if changes to the shape or adhesion of adjacent epithelial cells can impact the caliber of axons, we performed simultaneous time-lapse imaging of axons and the underlying basal cells starting at 28–31 hpf (Movie 4). We imaged basal cells using a marker of lipid microdomains under the control of the ΔNp63 promoter (Rasmussen et al., 2015; Rosa et al., 2023). One of the most dramatic and frequent perturbations to the RB axon environment occurs when basal cells round up for mitosis, during which the lipid reporter clearly highlights rounded cells against the field of surrounding, flat basal cells (Fig. 7*A*). We measured caliber at 1 µm intervals along the length of the axon, from a flat neighbor cell, onto the rounded cell, and onto the next flat neighbor cell (Fig. 7*B*). We found that segments of axon on the rounded cell were significantly thicker than segments of the same axon on the nondividing neighbor cells (caliber = 0.21 µm on rounded cell, 0.16 and 0.14 µm on nondividing neighbors; *p* = 0.025 and 0.00035, respectively; *n* = 114 measurement locations; *N* = 4 axons/fish; Fig. 7*C*; Table 1, g and h). This difference contrasts with the two nondividing neighbor groups, which did not have significantly different calibers, as expected (*p* = 0.059; Table 1, i). Although this observation does not distinguish between mechanical or signaling interactions as the driving factor for caliber changes, these data align with a model in which dynamic properties of the dividing cell may impact local axon caliber.

**Movie 4.** Time-lapse microscopy of RB axon in contact with a mitotic basal keratinocyte. Super-resolution time-lapse microscopy of an RB axon segment in contact with a skin cell that is undergoing mitosis. Morphological changes in both cells were visualized via fluorescent membrane tags. Magenta: RB neuron labeled with mRuby-caax. Green: basal keratinocyte labeled with egfp-plcδ-ph. Time interval, 5 min. Scale bar, 5 µm. [View online]

### Forces from surrounding cells may impact axon morphology

One reason why axons might be thicker on rounded cells could be due to changes in tension (Griswold et al., 2024; Pan et al., 2024). As a basal cell enters mitosis, it pulls its borders inward, becoming shorter in planar distance, and rounds up, creating a greater vertical distance (Fig. 7*D*). We used images with the clearest orthogonal projections to obtain estimates of how the dimensions of a dividing basal cell differ from its nondividing neighbors. Using these estimates, we calculated theoretical changes in axon length as the cell rounds up (Fig. 7*E*). To simplify these calculations, we assumed that the axon is fixed at the cell borders and runs in a straight path across the basal cell surface. We found that the rounding of basal cells resulted in a shorter axon path during division than when cells were flat (path length when round ≈ 0.78 × path length when flat). Assuming that axons are adhered to the nondividing neighboring cells, these estimates are consistent with the axon being under lower tension on the rounded cell than on a nondividing neighbor cell.

**Figure 8.**
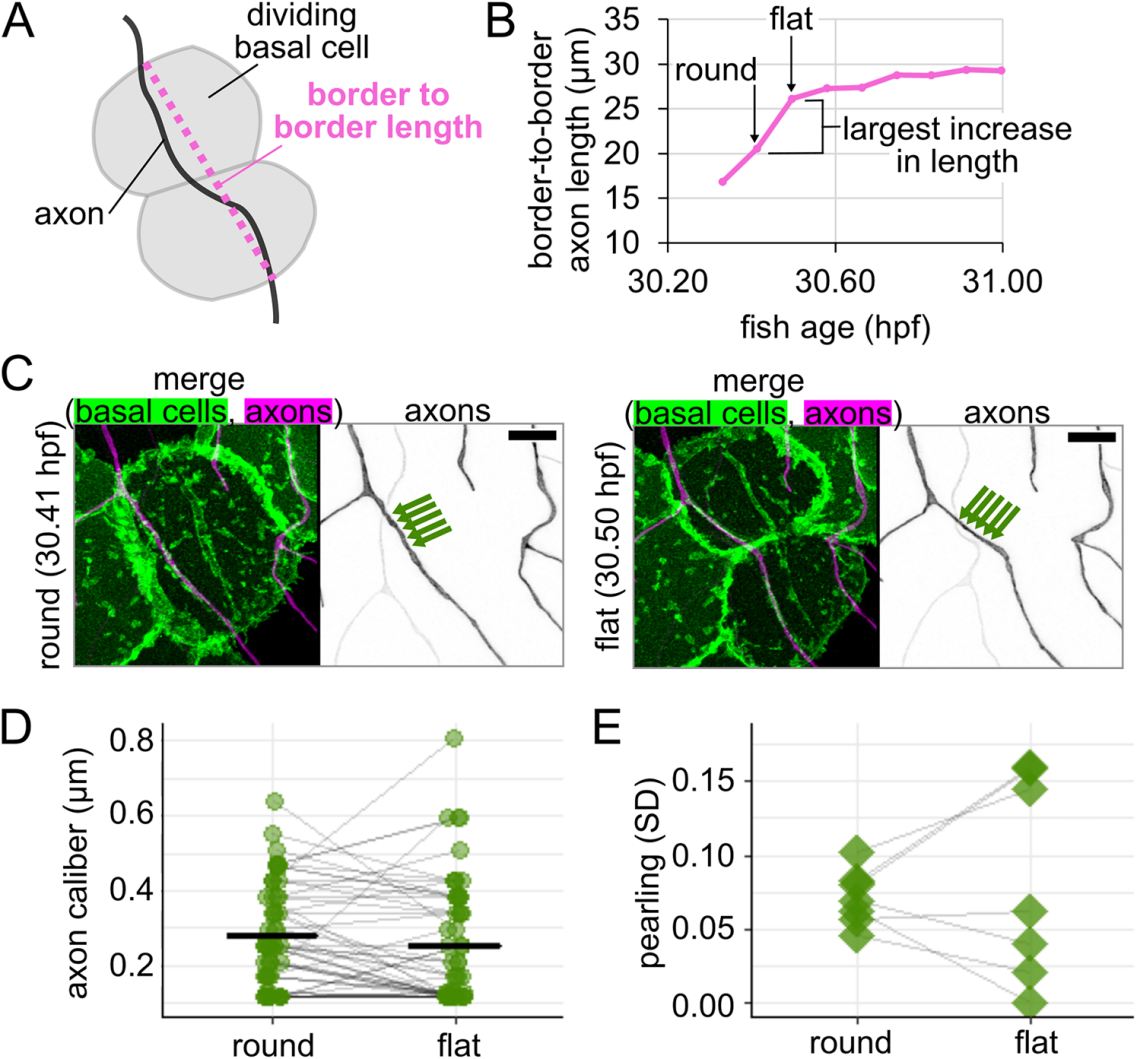
Changes to axon morphology as new basal epithelial cells flatten. ***A***, Diagram of how border-to-border length was measured for an axon on a dividing basal cell. ***B***, Example plot of changes in border-to-border axon length during time-lapse imaging for the axon shown in Figure 7*A*. Round and flat time points are defined as sequential points (5 min interval) during which the border-to-border length increases the most. ***C***, Images from time points highlighted in panel ***B***. Arrows: five example measurement locations. Scale bar, 5 µm. ***D***, Plot of axon caliber for all locations that remained on the dividing basal cell or its daughter cells, *n* = 54 measurement locations, *N* = 7 axons/fish. Gray lines connect data points from the same location. Black lines: mean values. ***E***, Plot of pearling, defined as SD across all locations, to assess morphology change for axons shown in panel ***D***, *N* = 7 axons/fish. Gray lines connect data points from the same axon.

Based on our observations of rounded cells and our estimates of length changes, we expected that axon caliber would decline as the daughter basal cells flatten. To test this prediction, we performed time-lapse imaging with 5 min intervals on the rounded cells, including those in Figure 7*C*, to see if axon caliber changes as the rounded cell divides and flattens into two daughter cells. For each time point, we measured the distance between the two points where the axon crossed the border of the dividing cell or its daughter cells (Fig. 8*A*). The two time points between which the change in length was greatest were selected, and the caliber of the axon on the rounded cell or its daughters was measured at 1 µm increments for those two time points (Fig. 8*B*,*C*). We paired measurements by location to assess if there was a significant drop in axon caliber between these two time points. Although axon caliber declined from the round to flat time point, on average, this subtle decrease did not reach statistical significance (caliber = 0.28 µm when the basal cell was round and 0.25 µm when the basal cell was flat, *p* = 0.17, *n* = 54 measurements, *N* = 7 axons/fish, Fig. 8*D*; Table 1, j). This contrasts with the portions of the axon that were on nondividing neighbor cells, where the difference was even smaller and was more likely to occur by random chance (*p* = 0.94, *n* = 37 measurements; Table 1, k). This result suggests that the decrease in average axon caliber as the underlying basal cell flattens is small and likely not meaningful.

Despite the lack of change in average caliber, visual assessment of the movies appeared to show a change in morphology during basal cell flattening (Figs. 7*A*, 8*C*). Tension and mechanical force may be important parameters in pearling (Bar-Ziv et al., 1999; Pullarkat et al., 2006; Griswold et al., 2024; Pan et al., 2024), so we wondered if thick regions (i.e., pearls) and thin regions (i.e., connectors) undergo different changes during basal cell division.

To assess morphological changes to axons during basal cell division, we calculated pearling as the standard deviation of measurements taken along the length of each axon for the rounded time point and for the flat time point. By pairing values from each movie, we found that one subset of axons became more pearled (increased SD) and the rest became less pearled (decreased SD), resulting in two groups of cellular responses to flattening (Fig. 8*E*). This heterogeneity might suggest that either (1) different responses arise from heterogeneity among the RB neuron population or (2) our 5 min intervals do not provide sufficient time resolution to capture cellular responses to tension, which may occur in <10 min (Sinha et al., 2011; Sitarska and Diz-Muñoz, 2020). Regardless of the reason, these results demonstrate that axon morphology changes with basal cell flattening, though additional complexities remain to be explored.

## Discussion

### Tapered architecture in branched sensory axons

We have found that the calibers of axon segments in the same RB neuron can be largely independent of one another, highly dynamic, and likely shaped by multiple determinants, including contact with epithelial cells. Variation in axon caliber had previously been observed along short stretches of fixed axons in rodent neurons (Greenberg et al., 1990; Griswold et al., 2024), in response to developmental changes to the neuron's environment and signaling (Sánchez et al., 1996; Kumar et al., 2005; Seidl et al., 2010; Seidl, 2014; Ford et al., 2015; Seidl and Rubel, 2016; Sinclair et al., 2017; Metzner et al., 2022; Bin et al., 2024; Nabel et al., 2024), and with forces on millimeters-length scales (Pan et al., 2024). Our results expand upon these findings by characterizing caliber variations in vivo across a branched axon, with fine timescale, and with local deformations caused by epithelial cell division.

The cutaneous axon endings of touch-sensing neurons, including trigeminal, dorsal root ganglion, and RB neurons, share characteristics with both dendrites and axons. Like dendrites, they detect stimuli from the periphery and relay signals toward the cell body, but, like axons, their microtubules are oriented plus-end-out (Shorey et al., 2021). We found that the branched axon arbors of RB neurons taper across the most proximal branch point, becoming narrower in caliber and cross-sectional area further from the soma (Fig. 4). This feature is often regarded as a characteristic of dendrites, not of axons (Craig and Banker, 1994; Jan and Jan, 2010). Mechanisms of signal relay from the periphery to the soma in RB neurons have not been fully elucidated. If RB neurons fire action potentials, then their tapering (Fig. 5) would favor afferent action potential conduction, as signals travel from the periphery toward the soma. In measuring the radius scaling ratio, we found it to be consistent with neurons optimized for power dissipation, which aligns well with a more graded, dendrite-like signal transduction (Desai-Chowdhry et al., 2022). Together, our findings are consistent with a model in which tapering may be most aligned with the direction of functional signal propagation rather than microtubule polarity. Whether our findings hold true for other axons, especially afferent axons, remains to be explored.

In comparing sister axon branches, we hypothesized that sister branches are related to each other, resulting from either cell-wide effects or a competition for cellular materials. Instead, we found that their calibers are largely uncorrelated with each other, despite being in close proximity and sharing a cytoplasm. Interestingly, we observed varying degrees of symmetry, ranging from pairs of sister branches with the same caliber to highly asymmetrical pairs, where one secondary branch is much thicker than the other. One reasonable hypothesis to explain this observation could be that branches of different symmetry form by different mechanisms. For example, highly asymmetric branches might have formed by collateral sprouting and symmetric branches by growth bifurcation. However, we found a full spectrum of symmetry values, not a bimodal distribution, arguing against this hypothesis (Fig. 3*D*,*E*). Moreover, though bifurcations are common in RB arbor outgrowth (Haynes et al., 2022), the first branch point often, if not always, forms as a collateral (Fig. 1*C*). Thus, collateral sprouting can give rise to segments of a variety of calibers within a few hours of formation, highlighting the malleability of axon caliber.

### Mechanisms of caliber variation over time and space

We found that caliber dynamicity changed over development (Fig. 6). Many developmental changes could account for a shift in dynamicity, including changes within the cell itself, such as cargo transport (Costa et al., 2020; Wang et al., 2020), or changes to the extracellular environment, such as axon ensheathment (O’Brien et al., 2012; Jiang et al., 2019; Rosa et al., 2023). Time-lapse imaging of axon caliber also revealed multiple qualitatively different types of dynamics (Fig. 5*A*–*D*), which we refer to as traveling pearls, focal inflation and deflation, segment widening and narrowing, and constriction points. This variety suggests that there are likely multiple contributors to variations in axon caliber, including both cell-intrinsic and cell-extrinsic mechanisms.

Cell-intrinsic determinants of axon caliber, such as the cytoskeleton, have been studied to varying degrees. One of the best-known caliber determinants is neurofilaments, a type of intermediate filament specific to the nervous system. Increased neurofilament abundance increases axon caliber in mammalian and bird neurons (Ohara et al., 1993; Sakaguchi et al., 1993; Eyer and Peterson, 1994; Zhu et al., 1997; Elder et al., 1998; Kriz et al., 2000; Sainio et al., 2021). Neurofilaments are also highly dynamic. Differences in transport, differential modifications, and dynamics (e.g., folding, severing, and annealing) of neurofilaments are all possible ways in which these filaments might contribute to both local regulation and axon caliber dynamics within a cell (Çolakoğlu and Brown, 2009; Boyer et al., 2022; Uchida et al., 2023).

Another cytoskeletal candidate for cell-intrinsic effectors of caliber variation is the more recently discovered membrane periodic skeleton (MPS), regularly spaced actin rings connected by spectrin tetramers that run the length of axons and dendrites (Xu et al., 2013; He et al., 2016). These rings expand when large cargo such as mitochondria and lysosomes pass through, altering myosin contractility changes caliber and retrograde trafficking (Leite et al., 2016; Costa et al., 2020; Wang et al., 2020). Observations of cargo transport through the MPS (Costa et al., 2020; Wang et al., 2020), as well as fixed observations that large cargo is often found within pearls (Greenberg et al., 1990), suggest that intracellular cargo transport along microtubules may also contribute to axon caliber dynamics. Griswold et al. (2024) demonstrated that myosin contractility can impact pearling. Furthermore, the MPS and the propensity of neurons to bead have been found to be protective against low-level mechanical forces (Krieg et al., 2017; Pan et al., 2024), highlighting MPS contractility as another potential source of caliber variations.

Other intracellular factors, such as importin beta (Bin et al., 2024), PI3K/AKT signaling (Kumar et al., 2005), and membrane composition (Griswold et al., 2024), also regulate axon caliber. Although some of these factors likely influence caliber by regulating the cytoskeleton (Bin et al., 2024), membrane composition is a noncytoskeletal, cell-intrinsic candidate for causing caliber variation. Griswold et al. (2024) found that membrane fluidity can change which size and shape of pearls that are most energetically favorable, thereby changing axon morphology. Thus, given the dynamic nature of caliber in vivo, changes to membrane composition could also change caliber dynamics.

Damage can also influence axon morphology. Markedly pearled or varicose morphologies, sometimes referred to as beading or swelling, often form in damaged axons (Kerschensteiner et al., 2005; Williams et al., 2014; Datar et al., 2019; Shao et al., 2020). Indeed, damaged trigeminal and RB axons, themselves, become dramatically more beaded just before degeneration (Martin et al., 2010). Mechanical forces below the level necessary to break axons have also been found to impact axon and dendrite pearling (Pan et al., 2024). Our observations of variation in caliber in this study were made in anesthetized, healthy embryos and are unlikely to result from axon damage, as neurons remained intact, even after time-lapse microscopy. Similarity between these phenomena in healthy and damaged axons may reflect related processes if the healthy malleability of axon caliber is pushed to extremes upon insult.

Although much attention has focused on potential intrinsic determinants for axon caliber variation, we considered the possibility that extrinsic influences from surrounding cells might affect axon caliber locally. Cell-extrinsic factors that influence axon caliber include target size (Voyvodic, 1989a) and distance (Seidl et al., 2010), neuronal function (Sinclair et al., 2017; Nabel et al., 2024), and mechanical forces at the millimeters-length scale (Pan et al., 2024). Each of these factors likely impacts the entire cell and is, therefore, unlikely to regulate caliber variation locally.

Our study focused on smaller deformations that occur on smaller length scales and timescales. Although myelination is thought to influence local axon caliber (Rydmark, 1981; Swärd et al., 1995; Seidl, 2014), questions remain about the direction and specific nature of this intercellular relationship (Friede, 1972; Sánchez et al., 1996; Bin et al., 2025). RB axon arbors are embedded in a developing epidermis, which undergoes growth and morphogenetic changes. RB axons span tens or even hundreds of micrometers in length and branch to cover large areas of the skin. Hence, each segment of an axon contacts different basal cells, which may each impact the local axon caliber independently.

Basal cells can undergo many morphological changes to alter the axon's environment. One important change is ensheathment (O’Brien et al., 2012; Fig. 1*A*), which increases between 1 and 2 dpf (Jiang et al., 2019; Rosa et al., 2023), possibly contributing to differences in dynamicity between these two developmental time points (Fig. 6*C*). The idea that ensheathment may alter axon caliber is further consistent with the observation that myelinated portions of an axon are thicker than the nodes of Ranvier (Rydmark, 1981; Swärd et al., 1995).

Another morphological change in basal cells is rounding for division, which we found changes axon caliber and morphology (Figs. 7, 8). We hypothesize that changes in tension caused by the movement of cells adjacent to axons are candidates for cell-extrinsic regulators of axon caliber. Division events likely vary in frequency throughout embryonic development, which may further contribute to developmental differences in caliber dynamicity (Fig. 6*C*). Additionally, axons may experience end-to-end stretch as embryos lengthen. Finally, although our studies were in embryonic zebrafish, when the environment surrounding these neurons is a rapidly expanding bilayered epidermis, dynamics are unlikely to cease in adulthood, since the stratified epidermis in both fish and mammals is constantly renewing as basal cells differentiate into higher strata. How caliber variations and dynamics differ in this stratified system remains to be explored. In conjunction with findings that myelination influences axon caliber (Rydmark, 1981; Swärd et al., 1995; Seidl, 2014; Walker et al., 2019; Ciocanel et al., 2020), our results support the model that lengthwise variation, local variations in average caliber, and caliber dynamics may be influenced, in part, by the behavior of surrounding cells.

## Synthesis

Reviewing Editor: Fabienne Poulain, University of South Carolina Decisions are customarily a result of the Reviewing Editor and the peer reviewers coming together and discussing their recommendations until a consensus is reached. When revisions are invited, a fact-based synthesis statement explaining their decision and outlining what is needed to prepare a revision will be listed below. The following reviewer(s) agreed to reveal their identity: Sarah Kucenas. Note: If this manuscript was transferred from JNeurosci and a decision was made to accept the manuscript without peer review, a brief statement to this effect will instead be what is listed below.

This manuscript reports a comprehensive descriptive study of the dynamics of axon caliber in zebrafish Rohon-Beard (RB) sensory neurons. Axon caliber is an important parameter of neuronal morphology with a direct influence on neuronal function. This study advances the field by introducing the RB neurons as a model system to study effects of axon caliber on neuronal function in intact, living embryos. This is important because, as the authors nicely show, axon caliber is dynamic and is influenced by the extracellular environment and surrounding cells. The authors show that actively dividing epithelial cells impact the axon caliber in the sensory neurons innervating the epithelium. Through their careful description of axon caliber dynamics in developing, branching axons in vivo, the authors lay the groundwork for future studies on mechanisms that regulate axon caliber and neuronal function.

The authors already have thoroughly responded to a previous review of this study and made edits accordingly. Reviewers only have a few minor suggestions:

1. The title uses the terminology "touch-sensory axons." To be more specific, using Rohon Beard neuron might be more appropriate given that other touch neurons, like DRGs, were not investigated.

2. The text mentions 7D, but there is no 7D present in the figure.

3. In figures 7 and 8, additional arrows would be helpful to see the exact locations of axons being measured with relation to the epithelial cells.

## Author Response

Response to Reviewers We are thrilled to hear that our manuscript is suitable for publication in eNeuro and appreciate the additional suggestions. We have revised the manuscript according to the recommendations, as described below.

We have also followed the Editor's guidance to add estimation statistics, which are summarized now in a Statistical table (Table 1).

------------------------------- 1. The title uses the terminology "touch-sensory axons." To be more specific, using Rohon Beard neuron might be more appropriate given that other touch neurons, like DRGs, were not investigated.

We thank the reviewers for this suggestion. This change has been made to the title.

2. The text mentions 7D, but there is no 7D present in the figure.

We apologize for any confusion. Panel 7D was present, but the label was not visible. This has been corrected.

3. In figures 7 and 8, additional arrows would be helpful to see the exact locations of axons being measured with relation to the epithelial cells.

We thank the reviewers for this suggestion. Arrows have been added to Fig 7A and 7B. Fig 8C previously contained arrowheads. We have changed these to arrows to improve clarity for readers.

## References

[B1] Bar-Ziv R, Tlusty T, Moses E, Safran SA, Bershadsky A (1999) Pearling in cells: a clue to understanding cell shape. Proc Natl Acad Sci U S A 96:10140–10145. 10.1073/pnas.96.18.10140 10468576 PMC17856

[B2] Bin JM, Suminaite D, Benito-Kwiecinski SK, Kegel L, Rubio-Brotons M, Early JJ, Soong D, Livesey MR, Poole RJ, Lyons DA (2024) Importin 13-dependent axon diameter growth regulates conduction speeds along myelinated CNS axons. Nat Commun 15:1790. 10.1038/s41467-024-45908-6 38413580 PMC10899189

[B3] Bin JM, et al. (2025) Developmental axon diameter growth of central nervous system axons does not depend on ensheathment or myelination by oligodendrocytes. bioRxivorg.

[B4] Bomont P (2021) The dazzling rise of neurofilaments: physiological functions and roles as biomarkers. Curr Opin Cell Biol 68:181–191. 10.1016/j.ceb.2020.10.011 33454158

[B5] Boyer NP, Julien J-P, Jung P, Brown A (2022) Neurofilament transport is bidirectional in vivo. eNeuro 9:1–20. 10.1523/ENEURO.0138-22.2022 35896389 PMC9410771

[B6] Brummer AB, Savage VM, Enquist BJ (2017) A general model for metabolic scaling in self-similar asymmetric networks. PLoS Comput Biol 13:e1005394. 10.1371/journal.pcbi.1005394 28319153 PMC5378416

[B7] Ciocanel M-V, Jung P, Brown A (2020) A mechanism for neurofilament transport acceleration through nodes of Ranvier. Mol Biol Cell 31:640–654. 10.1091/mbc.E19-09-0509 32023144 PMC7202067

[B8] Çolakoglu G, Brown A (2009) Intermediate filaments exchange subunits along their length and elongate by end-to-end annealing. J Cell Biol 185:769–777. 10.1083/jcb.200809166 19468066 PMC2711597

[B9] Costa AR, et al. (2020) The membrane periodic skeleton is an actomyosin network that regulates axonal diameter and conduction. Elife 9:e55471. 10.7554/eLife.55471 32195665 PMC7105375

[B10] Craig AM, Banker G (1994) Neuronal polarity. Annu Rev Neurosci 17:267–310. 10.1146/annurev.ne.17.030194.001411 8210176

[B11] Datar A, Ameeramja J, Bhat A, Srivastava R, Mishra A, Bernal R, Prost J, Callan-Jones A, Pullarkat PA (2019) The roles of microtubules and membrane tension in axonal beading, retraction, and atrophy. Biophys J 117:880–891. 10.1016/j.bpj.2019.07.046 31427070 PMC6731471

[B12] Desai-Chowdhry P, Brummer AB, Savage VM (2022) How axon and dendrite branching are guided by time, energy, and spatial constraints. Sci Rep 12:20810. 10.1038/s41598-022-24813-2 36460669 PMC9718790

[B13] Dollé J-P, Jaye A, Anderson SA, Ahmadzadeh H, Shenoy VB, Smith DH (2018) Newfound sex differences in axonal structure underlie differential outcomes from in vitro traumatic axonal injury. Exp Neurol 300:121–134. 10.1016/j.expneurol.2017.11.001 29104114 PMC6495524

[B14] Elder GA, Friedrich VL Jr, Kang C, Bosco P, Gourov A, Tu PH, Zhang B, Lee VM, Lazzarini RA (1998) Requirement of heavy neurofilament subunit in the development of axons with large calibers. J Cell Biol 143:195–205. 10.1083/jcb.143.1.195 9763431 PMC2132822

[B15] Eyer J, Peterson A (1994) Neurofilament-deficient axons and perikaryal aggregates in viable transgenic mice expressing a neurofilament-beta-galactosidase fusion protein. Neuron 12:389–405. 10.1016/0896-6273(94)90280-1 8110465

[B16] Faas FGA, Avramut MC, van den Berg BM, Mommaas AM, Koster AJ, Ravelli RBG (2012) Virtual nanoscopy: generation of ultra-large high resolution electron microscopy maps. J Cell Biol 198:457–469. 10.1083/jcb.201201140 22869601 PMC3413355

[B17] Ford MC, Alexandrova O, Cossell L, Stange-Marten A, Sinclair J, Kopp-Scheinpflug C, Pecka M, Attwell D, Grothe B (2015) Tuning of Ranvier node and internode properties in myelinated axons to adjust action potential timing. Nat Commun 6:8073. 10.1038/ncomms9073 26305015 PMC4560803

[B18] Friede RL (1972) Control of myelin formation by axon caliber (with a model of the control mechanism). J Comp Neurol 144:233–252. 10.1002/cne.901440207 5029134

[B19] Gau P, Poon J, Ufret-Vincenty C, Snelson CD, Gordon SE, Raible DW, Dhaka A (2013) The zebrafish ortholog of TRPV1 is required for heat-induced locomotion. J Neurosci 33:5249–5260. 10.1523/JNEUROSCI.5403-12.2013 23516290 PMC3893356

[B20] Greenberg MM, Leitao C, Trogadis J, Stevens JK (1990) Irregular geometries in normal unmyelinated axons: a 3D serial EM analysis. J Neurocytol 19:978–988. 10.1007/BF01186825 2292722

[B21] Griswold JM, et al. (2024) Membrane mechanics dictate axonal pearls-on-a-string morphology and function. Nat Neurosci 28:49–61. 10.1038/s41593-024-01813-1 39623218 PMC11706780

[B22] Haynes EM, Burnett KH, He J, Jean-Pierre MW, Jarzyna M, Eliceiri KW, Huisken J, Halloran MC (2022) KLC4 shapes axon arbors during development and mediates adult behavior. Elife 11:e74270. 10.7554/eLife.74270 36222498 PMC9596160

[B23] He J, et al. (2016) Prevalent presence of periodic actin-spectrin-based membrane skeleton in a broad range of neuronal cell types and animal species. Proc Natl Acad Sci U S A 113:6029–6034. 10.1073/pnas.1605707113 27162329 PMC4889411

[B24] Hursh JB (1939) Conduction velocity and diameter of nerve fibers. Am J Physiol 127:131–139. 10.1152/ajplegacy.1939.127.1.131

[B25] Jan Y-N, Jan LY (2010) Branching out: mechanisms of dendritic arborization. Nat Rev Neurosci 11:316–328. 10.1038/nrn2836 20404840 PMC3079328

[B26] Jiang N, et al. (2019) A conserved morphogenetic mechanism for epidermal ensheathment of nociceptive sensory neurites. Elife 8:e42455. 10.7554/eLife.42455 30855229 PMC6450671

[B27] Katz HR, Menelaou E, Hale ME (2021) Morphological and physiological properties of Rohon-Beard neurons along the zebrafish spinal cord. J Comp Neurol 529:1499–1515. 10.1002/cne.25033 32935362

[B28] Kerschensteiner M, Schwab ME, Lichtman JW, Misgeld T (2005) In vivo imaging of axonal degeneration and regeneration in the injured spinal cord. Nat Med 11:572–577. 10.1038/nm1229 15821747

[B29] Krieg M, Stühmer J, Cueva JG, Fetter R, Spilker K, Cremers D, Shen K, Dunn AR, Goodman MB (2017) Genetic defects in ß-spectrin and tau sensitize *C. elegans* axons to movement-induced damage via torque-tension coupling. Elife 6:e20172. 10.7554/eLife.20172 28098556 PMC5298879

[B30] Kriz J, Zhu Q, Julien JP, Padjen AL (2000) Electrophysiological properties of axons in mice lacking neurofilament subunit genes: disparity between conduction velocity and axon diameter in absence of NF-H. Brain Res 885:32–44. 10.1016/S0006-8993(00)02899-7 11121527

[B31] Kumar V, Zhang M-X, Swank MW, Kunz J, Wu G-Y (2005) Regulation of dendritic morphogenesis by Ras-PI3K-Akt-mTOR and Ras-MAPK signaling pathways. J Neurosci 25:11288–11299. 10.1523/JNEUROSCI.2284-05.2005 16339024 PMC6725910

[B32] Leite SC, Sampaio P, Sousa VF, Nogueira-Rodrigues J, Pinto-Costa R, Peters LL, Brites P, Sousa MM (2016) The actin-binding protein α-adducin is required for maintaining axon diameter. Cell Rep 15:490–498. 10.1016/j.celrep.2016.03.047 27068466 PMC4838511

[B33] Martin SM, O’Brien GS, Portera-Cailliau C, Sagasti A (2010) Wallerian degeneration of zebrafish trigeminal axons in the skin is required for regeneration and developmental pruning. Development 137:3985–3994. 10.1242/dev.053611 21041367 PMC2976282

[B34] Metzner K, et al. (2022) Age-dependent increase of cytoskeletal components in sensory axons in human skin. Front Cell Dev Biol 10:965382. 10.3389/fcell.2022.965382 36393849 PMC9664158

[B35] Nabel AL, Teich L, Wohlfrom H, Alexandrova O, Heß M, Pecka M, Grothe B (2024) Development of myelination and axon diameter for fast and precise action potential conductance. Glia 72:794–808. 10.1002/glia.24504 38174817

[B36] O’Brien GS, Rieger S, Wang F, Smolen GA, Gonzalez RE, Buchanan J, Sagasti A (2012) Coordinate development of skin cells and cutaneous sensory axons in zebrafish. J Comp Neurol 520:816–831. 10.1002/cne.22791 22020759 PMC4299821

[B37] Ohara O, Gahara Y, Miyake T, Teraoka H, Kitamura T (1993) Neurofilament deficiency in quail caused by nonsense mutation in neurofilament-L gene. J Cell Biol 121:387–395. 10.1083/jcb.121.2.387 8468353 PMC2200107

[B38] Palanca AMS, Lee S-L, Yee LE, Joe-Wong C, Trinh LA, Hiroyasu E, Husain M, Fraser SE, Pellegrini M, Sagasti A (2013) New transgenic reporters identify somatosensory neuron subtypes in larval zebrafish. Dev Neurobiol 73:152–167. 10.1002/dneu.22049 22865660 PMC3541445

[B39] Pan X, et al. (2024) Actomyosin-II protects axons from degeneration induced by mild mechanical stress. J Cell Biol 223:e202206046. 10.1083/jcb.202206046 38713825 PMC11076810

[B40] Perge JA, Niven JE, Mugnaini E, Balasubramanian V, Sterling P (2012) Why do axons differ in caliber? J Neurosci 32:626–638. 10.1523/JNEUROSCI.4254-11.2012 22238098 PMC3571697

[B41] Pullarkat PA, Dommersnes P, Fernández P, Joanny J-F, Ott A (2006) Osmotically driven shape transformations in axons. Phys Rev Lett 96:048104. 10.1103/PhysRevLett.96.048104 16486900

[B42] Rasmussen JP, Sack GS, Martin SM, Sagasti A (2015) Vertebrate epidermal cells are broad-specificity phagocytes that clear sensory axon debris. J Neurosci 35:559–570. 10.1523/JNEUROSCI.3613-14.2015 25589751 PMC4293411

[B43] Rosa JB, Nassman KY, Sagasti A (2023) Sensory axons induce epithelial lipid microdomain remodeling and determine the distribution of junctions in the epidermis. Mol Biol Cell 34:ar5. 10.1091/mbc.E22-09-0396 36322392 PMC9816649

[B44] Rydmark M (1981) Nodal axon diameter correlates linearly with internodal axon diameter in spinal roots of the cat. Neurosci Lett 24:247–250. 10.1016/0304-3940(81)90165-8 7279292

[B45] Sagasti A, Guido MR, Raible DW, Schier AF (2005) Repulsive interactions shape the morphologies and functional arrangement of zebrafish peripheral sensory arbors. Curr Biol 15:804–814. 10.1016/j.cub.2005.03.048 15886097

[B46] Sainio MT, et al. (2021) Neurofilament light regulates axon caliber, synaptic activity, and organelle trafficking in cultured human motor neurons. Front Cell Dev Biol 9:820105. 10.3389/fcell.2021.820105 35237613 PMC8883324

[B47] Sakaguchi T, Okada M, Kitamura T, Kawasaki K (1993) Reduced diameter and conduction velocity of myelinated fibers in the sciatic nerve of a neurofilament-deficient mutant quail. Neurosci Lett 153:65–68. 10.1016/0304-3940(93)90078-Y 8510825

[B48] Sánchez I, Hassinger L, Paskevich PA, Shine HD, Nixon RA (1996) Oligodendroglia regulate the regional expansion of axon caliber and local accumulation of neurofilaments during development independently of myelin formation. J Neurosci 16:5095–5105. 10.1523/JNEUROSCI.16-16-05095.1996 8756439 PMC4556347

[B49] Schindelin J, et al. (2012) Fiji: an open-source platform for biological-image analysis. Nat Methods 9:676–682. 10.1038/nmeth.2019 22743772 PMC3855844

[B51] Seidl AH (2014) Regulation of conduction time along axons. Neuroscience 276:126–134. 10.1016/j.neuroscience.2013.06.047 23820043 PMC3849146

[B50] Seidl AH, Rubel EW, Harris DM (2010) Mechanisms for adjusting interaural time differences to achieve binaural coincidence detection. J Neurosci 30:70–80. 10.1523/JNEUROSCI.3464-09.2010 20053889 PMC2822993

[B52] Seidl AH, Rubel EW (2016) Systematic and differential myelination of axon collaterals in the mammalian auditory brainstem: systematic and differential myelination of axon collaterals. Glia 64:487–494. 10.1002/glia.22941 26556176 PMC4752408

[B53] Shao X, Sørensen MH, Xia X, Fang C, Hui TH, Chang RCC, Chu Z, Lin Y (2020) Beading of injured axons driven by tension- and adhesion-regulated membrane shape instability. J R Soc Interface 17:20200331. 10.1098/rsif.2020.0331

[B54] Shorey M, Rao K, Stone MC, Mattie FJ, Sagasti A, Rolls MM (2021) Microtubule organization of vertebrate sensory neurons in vivo. Dev Biol 478:1–12. 10.1016/j.ydbio.2021.06.007 34147472 PMC8364508

[B55] Sinclair JL, Fischl MJ, Alexandrova O, Heβ M, Grothe B, Leibold C, Kopp-Scheinpflug C (2017) Sound-evoked activity influences myelination of brainstem axons in the trapezoid body. J Neurosci 37:8239–8255. 10.1523/JNEUROSCI.3728-16.2017 28760859 PMC5566870

[B56] Sinha B, et al. (2011) Cells respond to mechanical stress by rapid disassembly of caveolae. Cell 144:402–413. 10.1016/j.cell.2010.12.031 21295700 PMC3042189

[B57] Sitarska E, Diz-Muñoz A (2020) Pay attention to membrane tension: mechanobiology of the cell surface. Curr Opin Cell Biol 66:11–18. 10.1016/j.ceb.2020.04.001 32416466 PMC7594640

[B58] Swärd C, Berthold CH, Nilsson-Remahl I, Rydmark M (1995) Axonal constriction at Ranvier’s node increases during development. Neurosci Lett 190:159–162. 10.1016/0304-3940(95)11528-5 7637883

[B59] Taylor NJ, Wang L, Brown A (2012) Neurofilaments are flexible polymers that often fold and unfold, but they move in a fully extended configuration. Cytoskeleton 69:535–544. 10.1002/cm.21039 22693112 PMC3415975

[B60] Tuttle AM, Miller LN, Royer LJ, Wen H, Kelly JJ, Calistri NL, Heiser LM, Nechiporuk AV (2024) Single-cell analysis of Rohon-Beard neurons implicates Fgf signaling in axon maintenance and cell survival. J Neurosci 44:e1600232024. 38423763 10.1523/JNEUROSCI.1600-23.2024 PMC11026351

[B61] Uchida A, Peng J, Brown A (2023) Regulation of neurofilament length and transport by a dynamic cycle of phospho-dependent polymer severing and annealing. Mol Biol Cell 34:ar68. 10.1091/mbc.E23-01-0024 36989035 PMC10295484

[B62] Voyvodic JT (1989a) Target size regulates calibre and myelination of sympathetic axons. Nature 342:430–433. 10.1038/342430a0 2586612

[B63] Voyvodic JT (1989b) Peripheral target regulation of dendritic geometry in the rat superior cervical ganglion. J Neurosci 9:1997–2010. 10.1523/JNEUROSCI.09-06-01997.1989 2542483 PMC6569718

[B64] Walker CL, Uchida A, Li Y, Trivedi N, Fenn JD, Monsma PC, Lariviére RC, Julien J-P, Jung P, Brown A (2019) Local acceleration of neurofilament transport at nodes of Ranvier. J Neurosci 39:663–677. 10.1523/JNEUROSCI.2272-18.2018 30541916 PMC6343641

[B65] Wang F, Julien DP, Sagasti A (2013) Journey to the skin: somatosensory peripheral axon guidance and morphogenesis. Cell Adh Migr 7:388–394. 10.4161/cam.25000 23670092 PMC3739816

[B66] Wang T, et al. (2020) Radial contractility of actomyosin rings facilitates axonal trafficking and structural stability. J Cell Biol 219:e201902001. 10.1083/jcb.201902001 32182623 PMC7199852

[B67] Williams PR, Marincu B-N, Sorbara CD, Mahler CF, Schumacher A-M, Griesbeck O, Kerschensteiner M, Misgeld T (2014) A recoverable state of axon injury persists for hours after spinal cord contusion in vivo. Nat Commun 5:5683. 10.1038/ncomms6683 25511170

[B68] Xu K, Zhong G, Zhuang X (2013) Actin, spectrin, and associated proteins form a periodic cytoskeletal structure in axons. Science 339:452–456. 10.1126/science.1232251 23239625 PMC3815867

[B69] Zhu Q, Couillard-Després S, Julien JP (1997) Delayed maturation of regenerating myelinated axons in mice lacking neurofilaments. Exp Neurol 148:299–316. 10.1006/exnr.1997.6654 9398473

